# Downregulation of IFNG‐AS1 and UCHL1‐AS1 in Peripheral Blood Cells of Multiple Sclerosis Patients: IFNG‐AS1 as a Candidate Diagnostic Biomarker

**DOI:** 10.1155/ijog/7231094

**Published:** 2026-09-19

**Authors:** Ali Rajabi, Jeffrey D. Gross, Ali Samadi

**Affiliations:** ^1^ Department of Animal Biology, Faculty of Natural Sciences, University of Tabriz, Tabriz, Iran, tabrizu.ac.ir; ^2^ ReCELLebrate Regenerative Medicine Clinic, Henderson, Nevada, USA; ^3^ Department of Basic Sciences, School of Medicine, Bam University of Medical Sciences, Bam, Iran, mubam.ac.ir

**Keywords:** biomarker, IFNG-AS1, multiple sclerosis, real-time PCR, UCHL1-AS1

## Abstract

**Background:**

Multiple sclerosis (MS) is a neurodegenerative and inflammatory disease affecting gray and white matter in the brain. Due to the highly variable presentation of MS, making a reliable long‐term diagnosis based solely on initial clinical findings is extremely challenging. Long noncoding RNAs (lncRNAs) have been shown in recent research to have a role as prospective biomarkers that may offer data to forecast the onset and course of disease.

**Methods:**

A total of 100 healthy controls and 120 MS patients with 98 relapsing‐remitting (RR), 10 primary progressive (PP), and 12 secondary progressive (SP) cases were included in the blood sample collection. Gene expression was assessed with quantitative real‐time PCR. The receiver operating characteristic (ROC) curve analysis was employed to evaluate the potential for diagnostic analysis of lncRNA levels.

**Results:**

The expressions of IFNG‐AS1 and UCHL1‐AS1 were found to be significantly decreased (*p* < 0.0001) in patients compared to the control group. After adjustment for age using ANCOVA, IFNG‐AS1 expression remained significantly lower in MS patients than in healthy controls (*p* < 0.0001). IFNG‐AS1 may be considered a potential biomarker for MS diagnosis (AUC = 0.838).

**Conclusion:**

IFNG‐AS1 demonstrates promising diagnostic potential for MS, whereas UCHL1‐AS1 shows only modest discriminatory value. However, these findings are preliminary and require further validation with functional studies to confirm clinical utility.

## 1. Introduction

Multiple sclerosis (MS) is a central nervous system (CNS) demyelinating and chronic inflammatory disease that mostly affects the spinal cord, the brain, and the optic nerves [1]. Its clinical presentation is variable, making different disease subvariant patterns difficult to identify. The pathologic characteristic of MS is several localized regions of myelin loss within the nervous system [2]. The current global MS population is approximately 1.89 million individuals, with a steady increase over the past 30 years [3]. The modified McDonald criteria defined relapsing‐remitting (RRMS), primary progressive (PPMS), and secondary progressive (SPMS) as the three main subtypes of MS. Significant individual variation has been observed during the disease [4, 5]. A vast array of symptoms, including pain, exhaustion, abnormalities of vision and senses, difficulty moving, and cognitive deficits, are typical of 85% of patients with RRMS subtype when the disease initially appears. Although it can sometimes appear in children, MS usually first affects adults between the ages of 20 and 40. Additionally, females are three times more likely to have MS [6, 7]. Although MS pathophysiology has been extensively researched, little is known about the potentially essential roles that transcriptional regulation and genetic variants may play in genomic regulatory regions such as enhancers, promoters, and other DNA elements that control gene expression [8]. Having clinical insight into particular MS subtypes can help guide treatment and allow for better diagnostic information for both clinicians and patients.

Identifying biomarkers in order to help subclassify MS disease activity and progression is the focus of numerous studies [9, 10]. One group of these biomarkers, lncRNAs, has been shown to contain more than 200 nucleotides [11]. Although most lncRNAs′ functions remain unclear, numerous studies have identified the crucial roles that lncRNAs play in various biological processes, including posttranscriptional modification, chromatin remodeling, transcriptional coactivation, and suppression of protein translation. The functions of cell differentiation, embryonic development, and several diseases, including neurodegenerative disorders, are all impacted by lncRNAs [12, 13]. Notably, several lncRNAs were found to be dysregulated in MS patients′ peripheral blood mononuclear cells (PBMCs), indicating the potential involvement of these genes in this disease′s pathophysiology [14].

The gene IFNG‐AS1, also known as Tmevpg1 (Theiler′s murine encephalomyelitis virus persistence candidate gene 1) or NeST (nettoie *Salmonella* pas Theiler′s), is an lncRNA transcript that is unique in that it is situated on the antisense DNA strand, opposite to the gene that encodes interferon‐gamma (IFN‐*γ*). T helper 1 (Th1) cells produce IFN‐*γ* actively, which is linked to the coexpression of lncRNA IFNG‐AS1 with T‐bet (encoded by the *TBX21* gene). T‐bet is a transcription factor that drives Th1 differentiation and IFN‐*γ* expression. Th1 cells are a subset of CD4+ T cells that are inflammatory and produce IFN‐*γ*. This inflammatory cytokine protects against intracellular infections but is linked to autoimmune disorders like ITP (immune thrombocytopenia) and delayed‐type hypersensitivity [14–16]. Several autoimmune diseases have been linked to concurrent overexpression of IFNG‐AS1 and IFN‐*γ* [17]. Their upregulation suggests a positive correlation between IFNG‐AS1 expression and the proportion of Th1 cells, as well as with the transcript levels of T‐bet and IFNG, suggesting a coordinated role in enhancing Th1‐mediated immune responses [18].

The PARK5 gene, also known as ubiquitin carboxy‐terminal hydrolase L1 (UCHL1), is a highly abundant protein in the brain [19]. The protein controls ubiquitin turnover, essential for the nervous system′s development [6]. UCHL1‐AS1, a natural antisense transcript from the UCHL1 gene′s opposing strand, stabilizes the UCHL1 mRNA, assisting in maintaining a high amount of UCHL1 in cells. By forming RNA‐RNA duplexes, it protects the UCHL1 mRNA from degradation and enhances its translation. Sustaining high UCHL1 levels supports neuronal function and protein homeostasis, which is particularly important in neurodegenerative disease contexts, where loss of UCHL1 function is linked to impaired synaptic activity and protein clearance. It has been discovered that natural antisense transcripts regulate the expression of genes in both cis and trans, and both concordant and discordant ways at the epigenetic, translational, transcriptional, and posttranscriptional levels [20, 21].

This study is aimed at evaluating IFNG‐AS1 and UCHL1‐AS1 lncRNA dysregulation in blood samples from MS patients and healthy controls. The associations between these lncRNAs′ expression levels and the patients′ clinical characteristics, such as age, gender, MS subtypes, and family history, were investigated.

## 2. Materials and Methods

### 2.1. Study Population and Controls

This case‐control study included 120 MS patients diagnosed by experienced neurologists using the 2017 McDonald criteria and was conducted in accordance with the Declaration of Helsinki. Patients were classified into three subtypes: RRMS (*n* = 98), SPMS (*n* = 12), and PPMS (*n* = 10). Inclusion criteria required a confirmed MS diagnosis based on the 2017 revised McDonald criteria, no immunomodulatory or immunosuppressive treatment for at least 6 months, and no significant comorbidities (e.g., diabetes, cancer, or other autoimmune diseases). It is important to mention that sample collection was performed at the time of initial diagnostic workup, before confirmation of MS diagnosis. Exclusion criteria included recent infections, pregnancy, or neurological disorders other than MS. The control group consisted of 100 healthy individuals, matched for age and sex, with no history of neurological, autoimmune, or inflammatory disorders. All participants provided written informed consent, and the Ethics Committee of Bam University of Medical Sciences approved the study (approval number: IR.MUBAM.REC.1403.051).

### 2.2. Sampling and Extraction of RNA

Peripheral blood (5 mL) was collected in EDTA‐coated tubes. PBMCs were isolated using Ficoll‐Paque density gradient centrifugation (1200 × g, 20 min, 20°C). Total RNA was extracted using TRIzol Reagent (DNAbiotech, Iran) according to the manufacturer′s protocol. Briefly, 1 mL TRIzol was added to 1 × 10^6^ PBMCs, followed by chloroform phase separation (200 *μ*L chloroform, centrifugation at 12,000 × g, 15 min, 4°C). RNA was precipitated with isopropanol, washed with 75% ethanol, and resuspended in RNase‐free water. RNA integrity was confirmed by visualizing 28S and 18S rRNA bands on 1% agarose gel electrophoresis. RNA purity was assessed using a NanoDrop spectrophotometer (Thermo Fisher Scientific), with 260/280 and 260/230 nm ratios ranging from 1.8 to 2.0. Extracted RNA was stored at −80°C until analysis.

### 2.3. Synthesis of Complementary DNA (cDNA) and Quantitative RT‐PCR

The cDNA was synthesized from 1 *μ*g RNA using the Yektatajhiz cDNA Synthesis Kit (Iran) in a 20 *μ*L reaction, following the manufacturer′s instructions (reverse transcription at 42°C for 60 min, inactivation at 85°C for 5 min). The cDNA was stored at −20°C until it was used for PCR. Quantitative real‐time reverse transcription‐PCR (qRT‐PCR) was performed using Maxima SYBR‐Green qRT‐PCR Master Mix (Thermo Fisher Scientific) on a Rotor‐Gene Q system. Primers for IFNG‐AS1, UCHL1‐AS1, and *β*‐actin (Table 1) were designed using Gene Runner and Primer‐BLAST, with specificity confirmed via NCBI BLAST. Amplicon sizes were as follows: IFNG‐AS1 (194 bp), UCHL1‐AS1 (108 bp), and *β*‐actin (70 bp). PCR conditions included an initial denaturation step at 95°C for 3 min, followed by 40 cycles of denaturation at 95°C for 15 s, annealing at 60°C for 30 s, and extension at 60°C for 30 s. Melt‐curve analysis was performed for each reaction to confirm the specificity of the amplification products. To eliminate genomic DNA contamination, RNA samples were treated with DNase I (Thermo Fisher Scientific) prior to cDNA synthesis, according to the manufacturer′s protocol. No‐template controls were included in each run to exclude contamination. The housekeeping gene *β*‐actin was selected as the reference gene, and its stability was confirmed in our cohort using the geNorm and NormFinder algorithms (*M* − value < 0.5), in addition to prior validation studies [22]. Relative expression was calculated using the 2^−*Δ*Ct^ method, where *Δ*Ct=Ct_target gene − Ct_*β*‐actin.

**Table 1:** Sequences of primers.

Gene name	Primer sequence	Product size
IFNG‐AS1	Forward: ACGCTGGAGGAGAAGTCAGT	194 bp
	Reverse: TGTTAGCAGTTGGTGGGCTT	

UCHL1‐AS1	Forward: TGGAGACGGGATTTGTTGGT	108 bp
	Reverse: GGGATGGTGAAAGGATGGGTT	

*β*‐actin	Forward: CTGGAACGGTGAAGGTGACA	70 bp
	Reverse: GTCCTCGGCCACATTGTGAA	

### 2.4. Examining Statistics

The statistical analyses were conducted using SPSS Version 26 and GraphPad Prism. The independent *t*‐test was used to examine differences between the two groups, and the one‐way ANOVA analysis was employed to identify significant differences among the three independent groups. To adjust for the potential confounding effect of age, an analysis of covariance (ANCOVA) was performed and partial eta‐squared (*η* ^2^) was calculated as a measure of effect size. The Spearman correlation test was used to assess each patient′s level of lncRNA expression. The receiver operating characteristic (ROC) curve′s AUC (area under the curve) showed that LncRNA levels were a useful diagnostic biomarker for MS. The threshold for statistical significance was set at a *p* value of less than 0.05.

## 3. Results

### 3.1. Individuals′ Characteristics

A total of 120 MS patients (with a mean age of 39 and SD 10.23) and 100 healthy controls (with a mean age of 30 and SD 5.43) were included in this study to perform additional research. Table 2 provides the participants′ demographic information.

**Table 2:** The patients′ and the healthy controls′ demographic information and expression means of IFNG‐AS1 and UCHL1‐AS1.

Variables	MS patients (*n* = 120)	Healthy controls (*n* = 100)	Expression mean (±SD) IFNG‐AS1		*p*	Expression mean (±SD) UCHL1‐As1		*p*
			MS	Control		MS	Control	
Sex					0.892			0.805
Female	73	58	0.0157 (0.0259)	0.0431 (0.0338)		0.0420 (0.0675)	0.0925 (0.1167)	
Male	47	42	0.0126 (0.0145)	0.0475 (0.0309)		0.0379 (0.0561)	0.0656 (0.0772)	
Age					**0.009**			0.914
< 30	28	49	0.0116 (0.0146)	0.0420 (0.0296)		0.0368 (0.0647)	0.0684 (0.0870)	
30–40	39	46	0.0162 (0.0189)	0.0470 (0.0364)		0.0374 (0.0582)	0.0816 (0.0945)	
> 40	53	5	0.0147 (0.0194)	0.0543 (0.0217)		0.0445 (0.0665)	0.2004 (0.2211)	
Family history					0.707			0.9
Yes	22	—	0.0135 (0.0155)	—		0.0361 (0.0619)	—	
No	98	—	0.0147 (0.0188)	—		0.0414 (0.0636)	—	
MS types					0.447			0.325
RR	98	—	0.0154 (0.0194)	—		0.0442 (0.0679)	—	
SP	12	—	0.0094 (0.0090)	—		0.0165 (0.0164)	—	
PP	10	—	0.0108 (0.0119)	—		0.0319 (0.0403)	—	

*Note:* Bold value indicates a statistically significant difference (*p* < 0.05).

### 3.2. Deregulation of IFNG‐AS1 and UCHL1‐AS1 Expression in MS Patients

**Figure 1:**
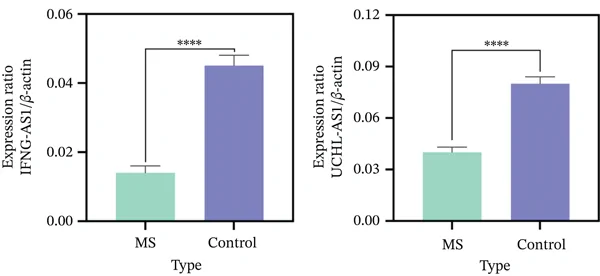
Relative expression levels of IFNG‐AS1 and UCHL1‐AS1 in peripheral blood mononuclear cells (PBMCs) of MS patients (*n* = 120) and healthy controls (*n* = 100), measured by quantitative real‐time PCR (qRT‐PCR) and normalized to *β*‐actin using the 2^−*Δ*Ct^ method. ∗∗∗∗*p* < 0.0001, independent *t*‐test.

Statistical analysis revealed significant downregulation of IFNG‐AS1 and UCHL1‐AS1 expression in MS patients compared to healthy controls (*p* < 0.0001) (Figure 1).

### 3.3. The Relationship Between Clinical Factors and Gene Expression

**Figure 2:**
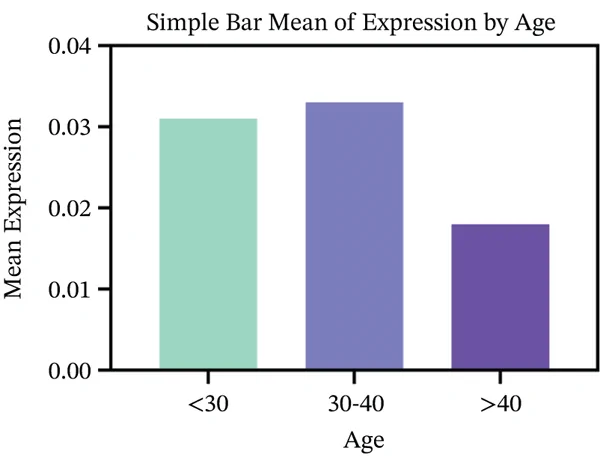
IFNG‐AS1 expression levels in PBMCs of MS patients and healthy controls stratified by age. Patients over 40 years of age exhibit significantly lower IFNG‐AS1 expression compared to the other two subgroups (*p* = 0.0007, independent *t*‐test).

No statistically significant variations were found between the two sex groups, MS types, and participant family backgrounds when comparing the age, gender, MS type, and family history attributes of patients and healthy groups. However, the subtype analysis (98 RRMS, 12 SPMS, and 10 PPMS) should be considered exploratory due to the small sample sizes in the SPMS and PPMS subgroups. As shown in Table 2, IFNG‐AS1 expression levels were significantly lower in MS patients compared to healthy individuals across all age groups (*p* value = 0.009). ANCOVA was performed to adjust for age as a potential confounding variable. After age adjustment, the mean expression of IFNG‐AS1 remained significantly lower in MS patients compared to healthy controls (adjusted mean difference = 0.0321, 95% CI: 0.0243–0.0399, *F* (1217) = 66.36, *p* < 0.0001, *η* ^2^ = 0.234), confirming that the downregulation of IFNG‐AS1 in patients is independent of age (Table 3). However, the > 40 age subgroup contained only 5 healthy controls compared to 53 MS patients, so the age‐stratified subgroup results should be interpreted with caution due to the small and unequal sample sizes in this subgroup. Additionally, the subgroups consist of participants under 30, those in their 30s and 40s, and those over 40, with the most pronounced decrease observed in patients over 40 years old (*p* value = 0.0007, Figure 2) but this finding requires confirmation in a larger, age‐matched cohort.

**Table 3:** ANCOVA results for IFNG‐AS1 expression adjusted for age.

Group	*n*	Raw *m* *e* *a* *n* ± *S* *D*	Adjusted mean	Adjusted mean difference	95% CI	*F* (df)	Degrees of freedom	*p*	*η* ^2^
Control	100	0.0450 ± 0.0326	0.0459	0.0321	[0.0243, 0.0399]	*F* (1, 217) = 66.36	217	< 0.0001	0.234
Control	100	0.0450 ± 0.0326	0.0459						

### 3.4. ROC Curve Analysis

**Figure 3:**
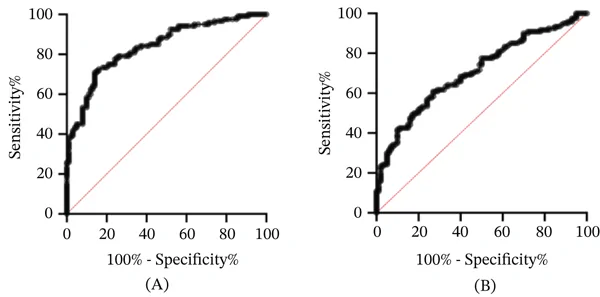
Receiver operating characteristic (ROC) curve analysis of (A) IFNG‐AS1 (AUC = 0.838, 95% CI: 0.7866–0.8899; sensitivity = 71.67*%*, specificity = 85*%*) and (B) UCHL1‐AS1 (AUC = 0.7073, 95% CI: 0.6397–0.7749; sensitivity = 71*%*, specificity = 64*%*) as diagnostic biomarkers for MS, based on qRT‐PCR data from PBMCs of MS patients (*n* = 120) and healthy controls (*n* = 100).

ROC curve analysis was used to evaluate the diagnostic potential of these lncRNAs. IFNG‐AS1 demonstrated strong diagnostic discriminatory ability with an AUC of 0.838 (95% CI: 0.7866–0.8899). Using the Youden index to select the optimal cutoff, IFNG‐AS1 showed a sensitivity of 71.67% and specificity of 85% at a cutoff value of < 0.01568. UCHL1‐AS1 showed modest diagnostic performance with an AUC of 0.7073 (95% CI: 0.6397–0.7749), sensitivity of 71%, and specificity of 64% at a cutoff value of < 0.02886 (Figure 3). These findings indicate that IFNG‐AS1 has promising potential as a diagnostic biomarker for MS, whereas UCHL1‐AS1 shows only moderate discriminatory value.

### 3.5. Correlations Analysis

The results of Spearman′s correlation analysis showed that there was no correlation between the expression levels of IFNG‐AS1 and UCHL1‐AS1 (*p* value = 0.66, *r* = 0.04).

## 4. Discussion

The disease known as MS, which often affects women more frequently and usually develops during the reproductive years, results in intermittently progressive neurological dysfunction. Some therapies guard against MS relapses, and lowering early neuroinflammation is believed to enhance long‐term outcomes. There is currently no biomarker that can help identify MS patient groups who would benefit from more aggressive therapies and predict a more severe course of their MS disease. lncRNAs are becoming known as possible disease biomarkers that may be useful in predicting the prognosis of MS [23].

Numerous studies in recent years have shown that MS patients have deregulated lncRNA [11, 12, 24]. Several lncRNAs, such as IFNG‐AS1‐003, RN7SK RNA, lnc‐DDIT4, LincR‐Epas1‐3’AS, Linc‐MAF‐4, and LincR‐Gng2‐5′AS, have been demonstrated to regulate the immune system [25]. Some are associated with several pathways of involvement, including PANDA, TUG1, and LincRNA‐p21, which are associated with the p53 tumor suppressor pathway and the cell cycle [5, 26, 27].

As mentioned by Li et al. [28], there may be a role for IFNG‐AS1 in the pathophysiology of autoimmune diseases because abnormal IFNG‐AS1 expression has been connected to several autoimmune disorders. One study found that patients with childhood ITP had elevated levels of IFNG‐AS1 in their peripheral blood, which can be targeted for therapy when necessary [16].

According to another study, there is an increase in the amount of IFNG mRNA and the proportion of circulating Th1 in people with Hashimoto′s thyroiditis (HT), another autoimmune disease. Furthermore, there was an upregulation of IFNG‐AS1 expression, which was favorably correlated with the expression of IFNG and the proportion of circulating Th1 cells [18].

On the other hand, high quantities of Th cells and related chemokines and cytokines in the cerebrospinal fluid (CSF) and CNS lesions of MS patients contribute to increased blood–brain barrier permeability. Interestingly, Th1, Th17, Th9, Th22, and Th1‐like Th17 cell numbers have all been linked to the MS pathogenesis [29].

According to a study, when RRMS patients′ expression levels of plasma IFNG‐AS1 were compared to those of healthy controls, they were significantly higher in relapse and remission [17], thereby demonstrating IFNG‐AS1 expression to poorer clinical outcomes.

The discrepancies in IFNG‐AS1 expression across studies may stem from several factors. First, sample type differences could play a role. Our study analyzed PBMCs, whereas Ghaiad et al. [17] measured plasma levels of IFNG‐AS1, which may reflect distinct aspects of lncRNA biology. However, PBMCs are the most commonly used source for RNA profiling due to their superior RNA stability and quality compared to whole blood samples. Second, disease phase variations are critical, as IFNG‐AS1 expression is elevated during both relapse and remission phases of RRMS but may differ in progressive MS subtypes or untreated patients, as in our cohort. Third, genetic and environmental factors specific to the geographical population tested may contribute, as Ganji et al. [30] also reported downregulated IFNG‐AS1 in a similar geography of RRMS patients, contrasting with findings in other populations [17]. These differences highlight the need for standardized sample types and disease phase stratification in future studies.

The significant age‐related differences in IFNG‐AS1 expression suggest a potential link to immunosenescence. Aging is associated with altered immune responses, including reduced Th1 cell activity and IFN‐*γ* production, which may lead to downregulation of IFNG‐AS1 expression [31]. This immunosenescence with age could exacerbate neuroinflammation and MS progression in older patients, as IFNG‐AS1 modulates IFN‐*γ*‐mediated immune responses [14]. After adjusting age difference between patients and control groups with ANCOVA test, we confirmed that IFNG‐AS1 expression levels were significantly lower in MS patients compared to healthy individuals across all age groups. Clinically, these findings underscore the importance of age‐stratified analyses in MS biomarker studies, suggesting that IFNG‐AS1 levels may reflect age‐dependent disease severity and therapeutic response.

Functionally, IFNG‐AS1 may modulate MS neuroinflammation by regulating IFN‐*γ* expression in Th1 cells, as its coexpression with T‐bet enhances IFN‐*γ* transcription [14, 30] . Downregulation of IFNG‐AS1 in MS patients may impair Th1‐mediated immune responses, potentially exacerbating neuroinflammatory damage. Similarly, UCHL1‐AS1 may influence neuronal integrity by stabilizing UCHL1 mRNA, which supports ubiquitin‐dependent protein clearance.

Its downregulation in MS patients suggests a disrupted neuroprotective mechanism, contributing to neurodegeneration. However, these hypotheses require direct experimental validation through further in vitro and in vivo studies, such as siRNA‐mediated knockdown of IFNG‐AS1 in Th1 cells or UCHL1‐AS1 overexpression in neuronal cell lines, could elucidate their mechanistic roles in MS pathogenesis and validate their therapeutic potential.

Relapsing phase MS patients had higher levels of IFNG‐AS1‐001 lncRNA in their PBMCs of 50 RRMS compared to healthy controls, whereas remitting phase MS patients had higher levels of IFNG‐AS1‐003 lncRNA. These findings were reported by Hosseini et al. [22]. The relationship between IFNG and IFNG‐AS1‐001 expression suggests that this lncRNA could be a promising target for MS treatment.

Children with autism spectrum disorder (ASD) had significantly higher amounts of IFNG and lower levels of IFNG‐AS1 expression compared to controls, as shown by Fallah et al. [32]. Additionally, they discovered a functional disruption in the IFNG/IFNG‐AS1 regulatory interaction network, which could contribute to the chronic inflammatory feature of ASD.

Carrieri et al. found that the UCHL1‐AS1 was markedly downregulated in in vivo and in vitro Parkinson′s disease (PD) mice. According to a study, UCHL1‐AS1 is mutated in a rare form of early‐onset familial PD. Moreover, UCHL1‐AS1 expression is regulated by NURR1, a transcription factor associated with the maintenance and maturation of dopaminergic cells; mutations in NURR1 have also been connected to PD [19].

Although UCHL1‐AS1 is normally located in the nucleus, it can be transferred to the cytoplasm in response to certain stressors, which increases the amount of UCHL1 protein and promotes the expression of UCHL1 mRNA. Since overexpression of UCHL1 protein has a neuroprotective effect, overexpression of UCHL1‐AS1 could be part of a mechanism for cell salvage [19, 33].

Furthermore, a different study discovered that Huntington′s disease was associated with upregulation of the lncRNA UCHL1‐AS1 [34, 35].

Increased expression of UCHL1, which may be helpful in neurodegenerative diseases. Utilizing UCHL1‐AS1‐based as a therapeutic strategy can be an innovative approach to treatment [36].

Our results are consistent with previous research associating IFNG‐AS1 and UCHL1‐AS1 with immune and neurodegenerative disorders. IFNG‐AS1 downregulation in MS aligns with reports in other inflammatory diseases such as ASD and HT, where its dysregulation contributes to altered Th1 cell responses. Similarly, low levels of UCHL1‐AS1 expression have been reported in PD models, supporting its role in maintaining neuronal health via UCHL1 regulation.

Despite the promising results, our investigation has several limitations that should be acknowledged. First, our data are derived from a single‐center cohort and lack external validation in an independent population, which limits the generalizability of our findings. Second, although we adjusted for age, we did not include other potential confounders such as disease duration, relapse rate at the time of sampling, or vitamin D levels, which may influence lncRNA expression. Third, the small sample sizes in the SPMS (*n* = 12) and PPMS (*n* = 10) subgroups mean that our subtype‐specific findings should be considered exploratory and require validation in larger cohorts. Fourth, age‐stratified subgroup analysis was limited by uneven group sizes, particularly the > 40 age group with only five controls, which may have affected the stability of comparisons in this subgroup. Given these limitations, IFNG‐AS1 should be considered a preliminary candidate biomarker for MS diagnosis, and further validation in multicenter, longitudinal studies with standardized protocols is required before clinical application.

In further studies, an age‐matched control group would also further validate IFNG‐AS1 role as biomarker. Moreover, because lncRNAs can affect the regulation of gene expression, measuring the relevant gene expression could provide crucial context to confirm suggested regulatory mechanism in future studies. Future studies with larger sample sizes could benefit from continuous age analysis and in vitro and in vivo experiments can also support the findings of this research.

## 5. Conclusion

The statistical analysis revealed a significant downregulation of IFNG‐AS1 and UCHL1‐AS1 expression in PBMCs of MS patients compared to the healthy control group (*p* value < 0.0001). After adjusting age differences using ANCOVA, there was significant downregulation in the expression level of IFNG‐AS1 between the patient and control age groups. The diagnostic values of these lncRNAs were ascertained using the ROC curve. With IFNG‐AS1′s AUC of 0.838 and UCHL1‐AS1′s AUC of 0.7073, IFNG‐AS1 emerges as a more promising diagnostic biomarker candidate for MS, whereas UCHL1‐AS1 may have limited value as standalone marker but could be considered in combination with other biomarkers. Although these lncRNAs show potential as components of a diagnostic panel, their clinical utility requires further validation in large‐scale, multicenter studies with standardized protocols and established cutoffs.

## Author Contributions

All authors contributed to the study conception and design. **Ali Samadi:** writing – review and editing, writing – original draft, supervision, methodology, formal analysis. **Ali Rajabi:** writing – original draft, writing – review and editing, methodology. **Jeffrey D. Gross:** writing – review and editing, methodology, formal analysis.

## Funding

This study was supported by Bam University of Medical Sciences, Bam, Iran (Grant No. 402000051).

## Ethics Statement

The Ethics Committee of Bam University of Medical Sciences approved this study (Ethical code # IR.MUBAM.REC.1403.051). The research also followed the tenets of the Declaration of Helsinki. This study was extracted from a research project that was conducted in Bam University of Medical Sciences. Additionally, ethical issues (including plagiarism, data fabrication, and double publication) have been completely observed by the authors.

## Consent

Written informed consent was obtained from all patients.

## Conflicts of Interest

The authors declare no conflicts of interest.

## Data Availability

The data generated or analyzed during the study is available from the corresponding author upon reasonable request.
